# A comprehensive safety analysis confirms rhabdomyolysis as an uncommon adverse reaction in patients treated with trabectedin

**DOI:** 10.1007/s00280-012-1864-4

**Published:** 2012-04-07

**Authors:** Federica Grosso, Maurizio D’Incalci, Mirela Cartoafa, Antonio Nieto, Carlos Fernández-Teruel, Vicente Alfaro, Pilar Lardelli, Elena Roy, Javier Gómez, Carmen Kahatt, Arturo Soto-Matos, Ian Judson

**Affiliations:** 1 SC Oncologia, SS Antonio and Biagio and C Arrigo General Hospital, Alessandria, Italy; 2 Department of Oncology, Mario Negri Institute for Pharmacological Research, Milan, Italy; 3 PharmaMar, Clinical R&D, Colmenar Viejo, Madrid, Spain; 4 Department of Medicine, Sarcoma Unit, Royal Marsden Hospital, London, UK

**Keywords:** Trabectedin, Rhabdomyolysis, Creatine phosphokinase

## Abstract

**Purpose:**

This analysis determined the incidence of serious rhabdomyolysis events reported during trabectedin treatment since the first phase I clinical trial in April 1996 up to September 2010.

**Methods:**

Search was done in the Yondelis^®^ Pharmacovigilance and Clinical Trials databases using a list of terms according to the Medical Dictionary for Regulatory Activities (MedDRA, v. 13.1), followed by a medical review of all cases retrieved. Total estimated sample was 10,841 patients: 2,789 from clinical trials; 3,926 from compassionate use programs; and 4,126 treated in the marketplace. Two groups were identified: (1) rhabdomyolysis and (2) clinically relevant creatine phosphokinase (CPK) increases without acute renal failure (ARF). Descriptive analysis included demographic, clinical/laboratory data, and contributing/confounding factors. Potential predictive factors were evaluated by multivariate stepwise logistic regression analysis. Possible changes of pharmacokinetics (PK) in patients with rhabdomyolysis were explored using a population PK model.

**Results:**

The global incidence of rhabdomyolysis was 0.7 %, and most cases occurred in Cycle 2 of treatment. The incidence of fatal cases was 0.3 %. None of the variables evaluated to detect potential risk factors of rhabdomyolysis were predictive. Additionally, CPK increases (without ARF) were detected in 0.4 % of patients as an incidental finding with good prognosis.

**Conclusions:**

Rhabdomyolysis is an uncommon event during trabectedin treatment. Multivariate analyses did not show any potential factor that could be predictive or represent a significantly higher risk of developing rhabdomyolysis. Nevertheless, close patient monitoring and adherence to drug administration guidelines may help to limit the incidence of this event.

## Introduction

Trabectedin (Yondelis^®^) is a marine-derived anticancer agent approved in 2007 as a single agent in the European Union for the treatment of soft tissue sarcoma (STS) after failure of standard-of-care chemotherapy (doxorubicin and/or ifosfamide) or for patients unsuited to receive these agents [9], and in 2009 for treatment of patients with relapsed, platinum-sensitive ovarian cancer in combination with pegylated liposomal doxorubicin (PLD; Caelyx^®^) [16]. Rhabdomyolysis has been reported as an uncommon adverse reaction associated with trabectedin treatment [2, 20, 22, 24]. Its etiology was unclear [12], as rhabdomyolysis was not observed during preclinical studies: only sporadic increased serum creatine phosphokinase (CPK) was found in repeat-dose studies in Cynomolgus monkeys, with no macro- or microscopic findings suggestive of trabectedin-induced rhabdomyolysis.

Rhabdomyolysis is an injury of skeletal muscle that releases potentially toxic muscle cell components (e.g., myoglobin, other intracellular proteins, and electrolytes) into the extracellular fluid and blood stream, which may result in renal damage [1, 21]. Rhabdomyolysis may have a number of causes, including direct trauma, excessive muscular activity, body-temperature extremes, muscle hypoxia, infections, metabolic and electrolyte disorders, endocrine disorders, connective tissue disorders, drugs, and toxins. Overall, the clinical features of rhabdomyolysis are quite variable and they can be summarized as muscular signs and symptoms (pain, weakness, tenderness, and contractures) and other symptoms (malaise, fatigue, fever, tachycardia, nausea, and vomiting). Rhabdomyolysis can result in life-threatening renal and multi-organ failure with hypovolemia, hyperkalemia, metabolic acidosis, disseminated intravascular coagulation, and compartment syndrome, secondary arrhythmias, or cardiac arrest [7, 14]. Biochemically, rhabdomyolysis is defined by marked blood CPK elevation [typically greater than 10 times the upper limit of normal (ULN)] with creatinine increase [6, 17]. CPK elevation is the most sensitive marker for skeletal muscle injury [5].

The aim of this analysis is to provide an updated and comprehensive summary of rhabdomyolysis events during treatment with trabectedin in adult patients with advanced solid tumors both as single agent or in combination with doxorubicin or PLD. Furthermore, we tried to identify potential predictive risk factors for this event.

## Materials and methods

The period analyzed was from the beginning of the first phase I clinical trial in April 1996 up to 17 September 2010, with a total estimated sample of 10,841 treated patients: 2,789 in clinical trials; 3,926 in compassionate use programs; and an estimation of 4,126 in the marketplace.

A search was carried out in both the Yondelis^®^ Pharmacovigilance and the Clinical Trials databases using a list of terms according to the Medical Dictionary for Regulatory Activities (MedDRA, v. 13.1). The preferred terms (PTs) of the standardized MedDRA query (SMQ) for rhabdomyolysis were used, as well as PTs from other MedDRA System Organ Class (SOCs) that could be related to rhabdomyolysis, or to the adverse events associated with trabectedin treatment. A medical review was performed for all cases retrieved, and 2 groups were identified: (1) rhabdomyolysis and (2) clinically relevant CPK increases without acute renal failure (ARF). The criteria for allocating a case to the rhabdomyolysis group were the following: cases reported as rhabdomyolysis, and cases with increased CPK (any grade) plus ARF, with or without symptoms of rhabdomyolysis or other diagnostic criteria [17]. The criteria for allocating a case to the CPK increases without ARF group were the following: CPK increases recorded as serious adverse events (any grade) in the Pharmacovigilance database, and CPK increases recorded as severe (grades 3 or 4) and with normal renal function or without available data on renal function in the Clinical Trials database. A descriptive analysis of these 2 groups of cases is shown here, including the main demographic, clinical and laboratory data, as well as contributing/confounding factors for rhabdomyolysis. All published cases of rhabdomyolysis with trabectedin up to the cutoff date for this analysis are included in the current assessment.

Potential predictive risk factors for rhabdomyolysis were explored. This analysis was performed in 2,321 patients treated in clinical trials from which all data necessary for cases and controls were available. Multivariate logistic regressions with stepwise variable selection were used to evaluate the possible relationship between the occurrence of rhabdomyolysis and categorical/continuous variables such as demography, previous medical history, concomitant treatments, indication for treatment, dose, and laboratory data of clinical interest.

The potential influence of trabectedin pharmacokinetic (PK) parameters in patients who developed rhabdomyolysis in clinical trials was explored using a population PK model [19]. Briefly, this open 4-compartment PK model consisted of a central plasma compartment and 3 peripheral compartments. Trabectedin was assumed to have linear and non-linear distribution from the central to the deep and shallow peripheral compartments, respectively. The model also included a catenary compartment representing a tissue compartment off the shallow peripheral compartment. The model was updated with the data from 900 patients with available PK data achieving parameters very similar to those already published. Multivariate logistic regression was used to evaluate the relationship between the occurrence of rhabdomyolysis and the main PK variables: clearance (CL), maximum plasma concentration (*C*_max_), and area under the curve (AUC) calculated from CL and dose administered.

## Results

### Rhabdomyolysis during treatment with trabectedin

Seventy-five of an estimated number of 10,841 treated patients had rhabdomyolysis (incidence of 0.7 %) (Table 1); these cases occurred in clinical trials (*n* = 26; incidence of 0.9 %), compassionate use programs (*n* = 26; 0.7 %), and in the marketplace (*n* = 23; 0.6 %).

**Table 1:** Cases of rhabdomyolysis and creatine phosphokinase increases (without acute renal failure) during treatment with trabectedin in cancer patients: demographic data

Total of cases (patients)	Rhabdomyolysis		CPK increases (without ARF)^a^	
	*n* = 75/10,841^b^	0.7 %	*n* = 48/10,841^b^	0.4 %
Age (years)^c^				
Median	55		58	
Range	21–83		14–80	
Gender (*n*, %)^c^				
Male	32	42.7 %	20	41.7 %
Female	41	54.7 %	27	56.3 %
Unknown	2	2.7 %	1	2.1 %

Indication	*n*/Total treated^d^	%	*n*/Total treated^d^	%
Bone sarcoma	–	–	2/280	0.7
Breast cancer	–	–	2/285	0.7
Endometrial cancer	2/65	3.1	–	–
Ovarian cancer	6/1,240	0.5	11/1,240	0.9
Soft tissue sarcoma	65/8,784	0.7	31/8,784	0.4
Unknown	2	–	2	–

Cutoff, September 17, 2010

The 75 patients with rhabdomyolysis were distributed among 15 different countries: Austria (*n* = 1), Belgium (*n* = 3), Brazil (*n* = 1), Canada (*n* = 4), China (*n* = 1), France (*n* = 7), Germany (*n* = 7), Greece (*n* = 1), India (*n* = 1), Italy (*n* = 5), Russia (*n* = 2), Spain (*n* = 11), Sweden (*n* = 1), United Kingdom (*n* = 7), and the USA (*n* = 23)

The 48 patients with CPK increases without ARF were distributed among 13 different countries: Brazil (*n* = 3), Canada (*n* = 3), France (*n* = 5), Germany (*n* = 1), Hong Kong (*n* = 1), Italy (*n* = 3), Korea (*n* = 1), Poland (*n* = 1), Russia (*n* = 1), Spain (*n* = 1), Sweden (*n* = 1), United Kingdom (*n* = 4), and the USA (*n* = 23)

*ARF* acute renal failure, *CPK* creatine phosphokinase, *NCI*-*CTCAE* National Cancer Institute Common Toxicity Criteria for Adverse Events

^a^CPK increases (without acute ARF) reported as serious adverse events (any grade) in the Pharmacovigilance database, or as severe (grade 3 or 4 according to NCI-CTCAE) in the Clinical Trials database

^b^The total number of patients treated in clinical trials, compassionate use, and in the marketplace estimate were 2,789, 3,926 and 4,126, respectively, for an approximate total number of 10,841 treated patients

^c^Data are shown for the 75 patients with rhabdomyolysis and the 48 patients with 58 CPK increases without ARF

^d^Based on rough estimate of total treated patients. The total estimate of 10,841 patients evaluated in this analysis also includes patients treated in other indications (e.g., lung, melanoma, etc.) in which rhabdomyolysis was not reported

Approximately half of these 75 patients (45.3 %) had one or more of the following contributive factors: incorrect posology; previous or concomitant liver disorders with potential effect on the drug metabolism; concomitant treatments (CYP3A4 inhibitors; other drugs known to produce rhabdomyolysis), and other rhabdomyolysis etiologies (Table 2).

**Table 2:** Possible contributing factors found in patients with rhabdomyolysis during treatment with trabectedin in cancer patients

	*n* = 75^a^	%
Incorrect treatment posology		
No reduction of dose when required	2	2.6
No delay of the cycle when required	1	1.3
Dose was increased with respect to that of previous cycle^b^	1	1.3
Possible effect on hepatic metabolism of the drug		
History of hepatic disorders (cirrhosis; chronic hepatitis C)	2	2.6
Hepatic metastases	5^c^	6.7
Acute hepatic disorder (portal vein thrombosis)	1	1.3
Important usual alcohol consumption	1	1.3
Concomitant administration of CYP3A4 inhibitors		
Amiodarone	2	2.6
Aprepitant	1	1.3
Clarithromycin	2	2.6
Diltiazem	1	1.3
Lercanidipine	1	1.3
Verapamil	2	1.3
Other confounding factors		
Concomitant statins (atorvastatin)	2	2.6
Concomitant Sinemet^®^ (carbidopa/levodopa)^d^	1	1.3
Hypothyroidism	4	5.3
Muscular trauma	1	1.3
Sepsis	5	6.7
Severe pneumonia	5	6.7

Cutoff, September 17, 2010

^a^Data are shown for the 75 patients with rhabdomyolysis. Patients could have more than one contributive factor

^b^Not allowed in the Summary of Product Characteristics of trabectedin

^c^All 5 patients with hepatic metastases were treated in clinical trials. Bulky (≥5 cm) liver metastases were found in 2 of these 5 patients. The incidence of rhabdomyolysis was similar in patients with liver metastases at baseline (0.8 %) with respect to those without liver metastases at baseline (0.9 %)

^d^This drug can induce serotoninergic syndrome, and this syndrome may secondarily induce rhabdomyolysis

The median age of patients with rhabdomyolysis was 55 years (range 21–83 years), and 41 of them (54.7 %) were females (Table 1). Most trabectedin clinical trials were conducted in patients with STS and ovarian cancer (958 and 687 of 2,321 treated patients with data on case/controls, respectively). Compassionate use programs were conducted in STS (3,929 patients), and STS and ovarian cancer are approved indications for trabectedin (~3,713 patient with STS and 413 patients with ovarian cancer of 4,126 treated patients in the marketplace). Therefore, the majority of patients who developed rhabdomyolysis were treated for STS (*n* = 65) at the dose approved for this indication 1.5 mg/m^2^ every 3 weeks (q3wk), followed by patients treated for ovarian cancer (*n* = 6), which is the second approved indication at the corresponding dose and schedule 1.1 mg/m^2^ q3wk. Nevertheless, the incidence of rhabdomyolysis in STS and ovarian cancer was similar. Rhabdomyolysis was reported as related to trabectedin treatment in 93.4 % of cases (Table 3).

**Table 3:** Cases of rhabdomyolysis and creatine phosphokinase increases (without acute renal failure) during treatment with trabectedin in cancer patients: cycles, regimens, relationship, concomitant medical conditions, and outcome

	Rhabdomyolysis		CPK increases (without ARF)^a^	
	*n* = 75^b^	%	*n* = 58^b^	%
Cycle of event onset				
1	10	13.3	7	12.1
2	43	57.3	12	20.7
3	10	13.3	8	13.8
4	2	2.7	4	6.9
5	2	2.7	6	10.3
>5	2	2.7	14	24.1
Unknown	6	8.0	7	12.1
Trabectedin regimen				
Weekly	–	–	4	6.9
0.38 mg/m^2^ d × 5 q3wk	1	1.3	–	–
0.4 mg/m^2^ q3wk	1	1.3	–	–
0.8 mg/m^2^ d1, 8, d15 q4wk	1	1.3	–	–
1.1 mg/m^2^ q3wk (plus PLD)	1	1.3	–	–
1.1 mg/m^2^ q3wk	2	2.7	–	–
1.2 mg/m^2^ q3wk	3	4.0	–	–
1.3 mg/m^2^ q3wk	3	4.0	–	–
1.5 mg/m^2^ q3wk	35	46.7	–	–
q3wk (dose unknown)	16	21.3	35	60.3
Unknown (dose and regimen)	12	16.0	19	32.8
Relationship with trabectedin treatment				
Related	70	93.4	50	86.2
Doubtful	1	1.3	2	3.4
Not assessed	3	4.0	–	–
Not related	–	–	3	5.2
Unknown	1	1.3	3	5.2
Concomitant medical conditions				
Neutropenia	53	70.7	25	43.1
Febrile neutropenia	20^c^	26.7	1^d^	1.7
Hepatic dysfunction	50^c^	66.7	30^d^	51.7
*Dialysis*	20	26.7	–	–
Outcome of the event				
Fatal	31	41.3	–	–
Recovered	25	33.3	40	69.0
Recovered with sequelae	4	5.3	1	1.7
Recovering	2	2.7	–	–
Not recovered	–	–	3	5.2
Unknown	13	17.3	14	24.1

Cutoff, September 17, 2010

*ARF* acute renal failure, *CPK* creatine phosphokinase, *d* day, *NCI*-*CTCAE* National Cancer Institute Common Toxicity Criteria for Adverse Events, *PLD* pegylated liposomal doxorubicin, *q3wk* every 3 weeks, *q4wk* every 4 weeks

^a^CPK increases (without acute ARF) reported as serious adverse events (any grade) in the Pharmacovigilance database, or as severe (grade 3 or 4 according to NCI-CTCAE) in the Clinical Trials database

^b^Data are shown for the 75 cases of rhabdomyolysis and the 58 cases of CPK increases without ARF. The total of patients treated for each variable was not available in marketplace and compassionate use in order to calculate the respective incidence

^c^Unknown: neutropenia (*n* = 18), febrile neutropenia (*n* = 23), and hepatic dysfunction (*n* = 25)

^d^Unknown: febrile neutropenia (*n* = 12) and hepatic dysfunction (*n* = 11)

The majority of cases were reported in Cycle 2 of trabectedin administration (*n* = 43; 57.3 %), and cases from Cycle 4 onwards were rare (*n* = 6; 8.0 %) (Table 3). The median day for CPK peak in rhabdomyolysis cases was Day 13.5 after last dose (range 2.0–33.0), and grade 3/4 CPK occurred in 33.3 % of patients.

Well-known adverse reactions associated with trabectedin treatment were identified as concomitant to rhabdomyolysis: neutropenia (any grade) in 70.7 % of the cases and liver function test alterations (any grade) in 66.7 % of the cases (Table 3).

Dialysis was performed in 20 cases (26.7 %) to treat the event (Table 3). In the remaining cases, corrective treatment usually consisted of intravenous hydration and occasionally urine alkalinization.

The prognosis of rhabdomyolysis depends on its early diagnosis and prompt management. In this series, 31 cases had a fatal outcome (incidence of 0.3 % with respect to the overall population of 10,841 patients). The time from onset of symptoms until diagnosis was not usually provided. Dialysis was performed in 12 (38.7 %) of the fatal cases, and not performed in 14 of them (45.2 %), in some cases due to end stage of the underlying malignancy or patient refusal (information was not provided in the other 5 fatal cases). Dialysis was not started at the time of diagnosis in 6 of the 12 fatal cases in which it was performed, being started only after clinical or laboratory deterioration (oliguria, anuria, metabolic acidosis, or important CPK elevation with respect to that at diagnosis).

### Cases of CPK increase without acute renal failure during treatment with trabectedin

Forty-eight of an estimated number of 10,841 treated patients (incidence of 0.4 %) (Table 1) had 58 events of clinically relevant CPK increase without ARF. Distribution by grade was as follows: grade 2, 5.2 %; grade 3, 43.1 %; grade 4, 34.5 %, and unknown grade, 17.2 %. All patients had normal renal function and no relevant clinical impact. Cardiac events were ruled out as a cause of CPK elevation. Muscular symptoms were present in only 13 patients (27.1 %), and important muscular effort during treatment was reported as a confounding factor in 2 patients.

The median age of these 48 patients was 58 years (range 14–80 years), and 27 of them (56.3 %) were females. Most cases were reported in patients treated for STS and ovarian cancer (*n* = 31 and *n* = 11, respectively), which are the 2 most evaluated indications for trabectedin, and 35 cases (60.3 %) occurred in the q3wk regimens (Table 3).

About half of these CPK increases occurred during the first 3 cycles of trabectedin (*n* = 27; 46.6 %) (Table 3). The median day for CPK peak was Day 18.0 after last cycle administration (range 2.0–34.0).

With respect to concomitant adverse reactions of trabectedin, neutropenia and liver function test alterations (any grade) were present in 43.1 and 51.7 % of the CPK increases without ARF, respectively (Table 3).

No patient required dialysis and the prognosis was generally good: 70.7 % of CPK increases recovered, 5.2 % did not recover at the moment of the report, and information was not provided in 24.1 % (Table 3).

### Multivariate analysis of cases of rhabdomyolysis from clinical trials

Twenty cases of rhabdomyolysis were observed in 2,321 patients treated in clinical trials with data of controls (patients included in clinical trials who did not develop rhabdomyolysis) for the analysis of predictive variables. Analyses were performed to determine potential risk factors; these included demographic data, indication, dose, prior history (hepatitis, liver metastasis, fatigue, myalgia, weakness), concomitant treatment, and laboratory status. Two multivariate logistic regression, stepwise selection models were evaluated. The first model included all cases (*n* = 20) and explored all the aforementioned covariates, baseline laboratory status, and laboratory data prior to the occurrence of rhabdomyolysis. The second model included cases of rhabdomyolysis that occurred in Cycle 2 and onwards (*n* = 17) and explored all covariates, with worst laboratory disorders reported in Cycle 1 (Table 4).

**Table 4:** Covariates selected in multivariate logistic regression (stepwise variable selection) analyses of rhabdomyolysis cases occurred in clinical trials with trabectedin in cancer patients

Parameter	Class	*p* Value	OR (95 % CI)
*Covariates selected when including all 20 rhabdomyolysis cases found in clinical trials*			
Theoretical trabectedin dose per week (μg/week)	–	0.0270	1.008 (1.001–1.016)
*Covariates selected when including rhabdomyolysis cases occurred from Cycle 2 onwards (n* = *17) and peak/nadirs of laboratory data occurred in Cycle 1 in clinical trials*			
Myelosuppression	Grade 0–2 versus grade 3–4	<0.0001	0.078 (0.024–0.258)
Transaminases increase	Grade 0–2 versus grade 3–4	0.0334	0.331 (0.120–0.917)

The constant was statistically significant in the two models

Degree of freedom was 1 for all selected covariates

*CI* confidence interval, *OR* odds ratio

The first model selected only one covariate: the theoretical trabectedin dose per week, with an odds ratio (OR) = 1.008 (95 % CI 1.001–1.016). According to this multivariate model, the probability for non-occurrence of rhabdomyolysis with trabectedin at the recommended dose for the regimen approved in STS (1.5 mg/m^2^ q3wk; i.e., 0.5 mg per week) is 98.7 % and with the regimen approved in ovarian cancer (1.1 mg/m^2^ q3wk; i.e., 0.37 mg per week; in combination with PLD) is 99.6 %.

The second model selected 2 covariates: myelosuppression (neutropenia and thombocytopenia) in Cycle 1 (OR = 0.078; 95 % CI 0.024–0.258), and transaminase elevation (ALT or AST) in Cycle 1 (OR = 0.331; 95 % CI 0.120–0.917) (Table 4). The probability of developing rhabdomyolysis in Cycle 2 or onwards was higher in those patients who had both grade 3/4 myelosuppression and transaminase elevation in Cycle 1. This underlines the need to modify the dose of trabectedin in subsequent cycles if significant liver impairment is observed or does not resolve prior to repeat dosing, as recommended in the Summary of Product Characteristics of trabectedin. These results also support that strict laboratory controls, fulfillment of criteria for treatment delay or continuation, and dose adjustment guidelines are extremely important to avoid unacceptable toxicities.

**Fig. 1:**
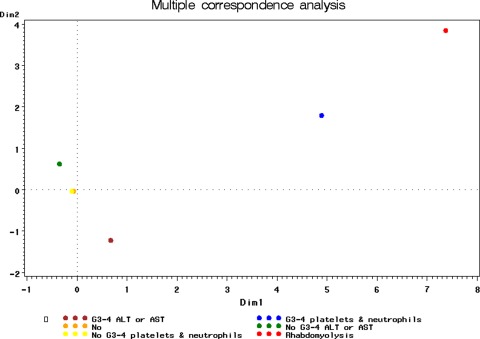
Multiple correspondence analysis between grade 3/4 myelotoxicity (neutrophils and platelets) and grade 3/4 transaminases occurred in Cycle 1 and occurrence of rhabdomyolysis from Cycle 2 onwards in clinical trials evaluating trabectedin

However, the model failed to identify definite predisposing factors for rhabdomyolysis. The spatial distribution according to a multiple correspondence analysis of the values of the 2 variables included in the multivariate logistic regression model (Fig. 1) accurately predicted the absence of rhabdomyolysis from Cycle 2 onwards in those cases without grade 3/4 myelotoxicity or transaminases increases in Cycle 1 (i.e., the “no grade 3/4” categories were grouped far from the “rhabdomyolysis” area). Nevertheless, the model could not predict either the occurrence of rhabdomyolysis in the presence of one or several of the aforementioned severe disorders (grade 3/4 laboratory abnormalities).

### Cases of rhabdomyolysis and pharmacokinetic analysis

**Fig. 2:**
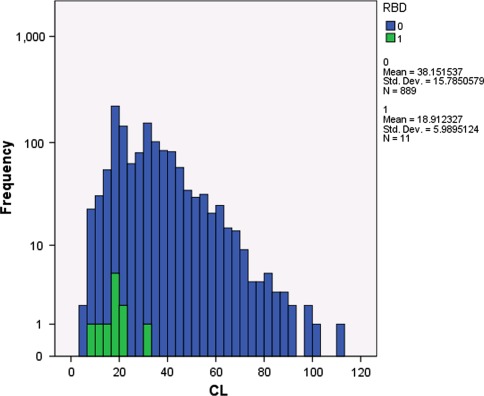
Frequencies for clearance (CL) in presence/absence of rhabdomyolysis. RBD = 0 (*blue histograms*) = absence of rhabdomyolysis. RBD = 1 (*green histograms*) = presence of rhabdomyolysis

The main PK variables [CL, *C*_max_, and AUC (population)] were explored in relationship with the occurrence of rhabdomyolysis (Table 5). A total of 11 of 900 evaluable patients with available PK data had rhabdomyolysis. In a multivariate logistic regression analysis, only the CL resulted significant (*p* < 0.0001) (i.e., higher probability of rhabdomyolysis in case of lower trabectedin CL), but was not identified as a risk factor, since most patients with low CL did not develop rhabdomyolysis (Fig. 2). In addition, the exposure PK parameters evaluated (*C*_max_ and AUC) did not show any significant relationship with the development of this event.

**Table 5:** Main trabectedin pharmacokinetic parameters in absence or presence of rhabdomyolysis

Rhabdomyolysis^a^	CL (l/h)	*C*_max_ (ng/ml)	AUC (h ng/ml)
Yes			
*n*	11	11	11
Mean	18.91	8.49	145.29
SD	5.99	10.08	71.66
Median	19.12	6.63	135.73
Min–Max	8.80–32.94	0.80–31.10	26.93–320.43
No			
*n*	889	889	889
Mean	38.15	7.71	59.28
SD	15.79	25.98	45.14
Median	36.16	3.83	48.20
Min–Max	4.50–110.07	0.04–487.97	1.03–395.03
Multivariate logistic regression analysis^b^			
*p* Value	<0.0001	0.3837	0.6738
OR (95 % CI)	0.845 (0.819–0.871)	–	–

*AUC* area under the curve, *C*_max_ maximum plasma concentration, *CI* confidence interval, *CL* clearance, *Max* maximum, *Mean* arithmetic mean, *Min* minimum, *OR* odds ratio, *PK* pharmacokinetic, *SD* standard deviation

^a^In those cases with PK data available in more than one cycle (first or second), only one PK profile per patient was selected for matching the rhabdomyolysis cases

^b^No statistically significant association between AUC and *C*_max_ of trabectedin and the occurrence of rhabdomyolysis was observed

## Discussion

This comprehensive safety analysis shows rhabdomyolysis to be an uncommon event during trabectedin treatment, with an overall incidence of 0.7 %. Apart from the treatment with trabectedin, confounding factors for the development of rhabdomyolysis were identified: concomitant treatments (atorvastatin, carbidopa/levodopa), hypothyroidism, muscular trauma, or infection (sepsis and severe pneumonia).

Rhabdomyolysis usually occurred during the first 2 cycles of treatment (53 of 75; 70.7 %), with only 2 cases after >5 cycles, which rules out a cumulative adverse effect of trabectedin. In this patient population, rhabdomyolysis was commonly accompanied by the presence of neutropenia and abnormal liver function tests, which suggests that this adverse reaction could be the result of intolerance to trabectedin. Multivariate analyses showed a higher probability of rhabdomyolysis in Cycle 2 or onwards in patients with concomitant grade 3/4 neutropenia and thrombocytopenia, and grade 3/4 transaminase increase in the first treatment cycle, but these disorders were not identified as risk or predictive factors of rhabdomyolysis, and they rather represented co-morbidities, which support the hypothesis of an uncommon trabectedin idiosyncratic reaction in patients who develop rhabdomyolysis, which may predispose them to develop other severe adverse reactions. In fact, transaminases increases are common in the setting of rhabdomyolysis, regardless of etiology. A retrospective review of 215 cases of rhabdomyolysis with CPK ≥ 1,000 IU/l and normal liver function showed abnormal AST in 93.1 % (95 % CI 88.7–95.8 %) and abnormal ALT in 75.0 % (95 % CI 68.7–80.2 %) of these patients [23]. Rhabdomyolysis can be further complicated with sepsis and multi-organ failure; then, neutropenia may also be a frequent concomitant adverse event [10, 15].

These results also support that appropriate laboratory controls and fulfillment of criteria for continuation of treatment and dose adjustment guidelines are extremely important to prevent the development of severe adverse reactions. In 2003, a retrospective evaluation based on data from early clinical trials suggested that premedication with dexamethasone improved hepatic tolerability to trabectedin, and since then, premedication with corticosteroids has been mandatory [4, 11, 13, 18]. In addition, close monitoring of hematological parameters, liver function and CPK were recommended weekly during the first two cycles. Criteria for treatment continuation or dose adjustment according to these laboratory parameters were implemented. As a result, the current analysis shows a decrease in the incidence of rhabdomyolysis in clinical trials from 1.0 % prior to 2003 to 0.7 % since 2003. An exponential regression to adjust the percentage of rhabdomyolysis cases per year up to the last available information was performed. A statistically negative independent parameter (*p* = 0.0139) was associated indicating that the incidence of this adverse event has decreased throughout the years. In addition, 4 of the rhabdomyolysis cases for which full information was available occurred in patients in whom criteria for re-treatment and dose adjustment were not followed according to current trabectedin administration guidelines.

Multivariate analyses showed the theoretical trabectedin dose per week to be statistically significant. The PK analysis showed no apparent relationship between drug exposure parameters (*C*_max_ and AUC) and occurrence of rhabdomyolysis but the logistic model of PK analysis showed that low CL was related. However, further PK predictive analysis showed that only a very small percentage of patients with low CL from the analyzed sample actually developed rhabdomyolysis. A significant number of patients with PK available for the present analysis were treated in phase I clinical trials, in which the dose administered varied significantly (from 0.024 to 1.8 mg/m²). This fact may explain why a relationship with CL was found, but not with AUC. Low trabectedin CL could reflect a condition in the patient (liver impairment, protein levels, polymorphisms affecting transporters) that had more impact on the probability of developing rhabdomyolysis than the trabectedin AUC itself, for example, by reflecting tissue, rather than plasma exposure. Therefore, it seems that a non-predictable individual sensitivity (e.g., an idiosyncratic reaction to the drug) could lead to this rare event.

Trabectedin is eliminated through hepatic metabolism and CYP3A4 is the principal responsible enzyme. As a high number of drugs are also metabolized by this enzyme, interactions with multiple concomitant drugs could arise. According to in vitro data, trabectedin exposure could be increased in the presence of CYP3A4 inhibitor [3]. Therefore, co-administration of trabectedin with potent CYP34A inhibitors should be avoided or, if this is not possible, closer patient monitoring is required. In this analysis, 8 patients with rhabdomyolysis had concomitant administration of CYP3A4 inhibitors (verapamil, diltiazem, amiodarone, lercanidipine, aprepitant, and clarithromycin). Additionally, 8 patients had prior or concomitant liver conditions that could have affected the hepatic metabolism of trabectedin.

Rhabdomyolysis is a treatable disorder, and patient prognosis depends on early recognition and immediate treatment, such as parenteral hydration, urine alkalinization, and dialysis [5, 14]. In this analysis, dialysis was reported in 26.7 % of cases. As most patients had advanced-stage cancer, a high number of them refused treatment for this event or they were not considered candidates for treatment, which likely contributed in some cases to the fatal outcome. Late treatment start once the event had worsened may have also contributed to fatal prognosis. Treatment with trabectedin should be discontinued when rhabdomyolysis is diagnosed.

Creatine phosphokinase increases have been usually observed in 20–30 % of patients treated with trabectedin [4, 8, 9], although most of them are grade 1/2 laboratory abnormalities without clinical significance. In order to take a conservative approach, the present analysis also included cases reporting serum CPK increase in the absence of ARF and considered clinically relevant, that is, severe (grade 3/4) and serious (any grade) adverse events. Clinically relevant CPK increases were also uncommon (0.4 % in the total of treated patients), and their onset appeared during the first 3 cycles of treatment in almost half of the cases. These events were associated with neutropenia in 41.3 % of cases, and abnormal liver function was reported in about half of cases (51.7 %). Overall, this appeared as an incidental finding, without clinical impact, and with a good prognosis.

In conclusion, this comprehensive safety analysis confirms rhabdomyolysis and clinically relevant (severe or serious) CPK increases without ARF to be uncommon events associated with trabectedin treatment. Multivariate and PK analyses did not show any predictive or high risk factor of developing rhabdomyolysis, thus suggesting the event as a possible idiosyncratic reaction. Prompt diagnosis and management of rhabdomyolysis is crucial for its resolution. Close patient monitoring, especially during the first 3 cycles, and adequate treatment adjustment in cases of severe myelosuppression and liver dysfunction are mandatory. Implementation of corticosteroid pre-treatment and close monitoring with dose adjustments according to myelosuppression, liver function abnormalities, and CPK levels have contributed to a reduction in the incidence of rhabdomyolysis and are currently included in the guidelines for trabectedin administration.

## References

[1] Bosch X, Poch E, Grau JM (2009). Rhabdomyolysis and acute kidney injury. N Engl J Med.

[2] Brain EG (2002). Safety and efficacy of ET-743: the French experience. Anticancer Drugs.

[3] Brandon EF, Sparidans RW, Guijt KJ, Lowenthal S, Meijerman I, Beijnen JH, Schellens JH (2006). In vitro characterization of the human biotransformation and CYP reaction phenotype of ET-743 (Yondelis, Trabectidin), a novel marine anti-cancer drug. Invest New Drugs.

[4] Carter NJ, Keam SJ (2007). Trabectedin: a review of its use in the management of soft tissue sarcoma and ovarian cancer. Drugs.

[5] Cervellin G, Comelli I, Lippi G (2010). Rhabdomyolysis: historical background, clinical, diagnostic and therapeutic features. Clin Chem Lab Med.

[6] Cziraky MJ, Willey VJ, McKenney JM, Kamat SA, Fisher MD, Guyton JR, Jacobson TA, Davidson MH (2006). Statin safety: an assessment using an administrative claims database. Am J Cardiol.

[7] Chatzizisis YS, Misirli G, Hatzitolios AI, Giannoglou GD (2008). The syndrome of rhabdomyolysis: complications and treatment. Eur J Intern Med.

[8] Del Campo JM, Roszak A, Bidzinski M, Ciuleanu TE, Hogberg T, Wojtukiewicz MZ, Poveda A, Boman K, Westermann AM, Lebedinsky C (2009). Phase II randomized study of trabectedin given as two different every 3 weeks dose schedules (1.5 mg/m^2^ 24 h or 1.3 mg/m^2^ 3 h) to patients with relapsed, platinum-sensitive, advanced ovarian cancer. Ann Oncol.

[9] Demetri GD, Chawla SP, von Mehren M, Ritch P, Baker LH, Blay JY, Hande KR, Keohan ML, Samuels BL, Schuetze S, Lebedinsky C, Elsayed YA, Izquierdo MA, Gomez J, Park YC, Le Cesne A (2009). Efficacy and safety of trabectedin in patients with advanced or metastatic liposarcoma or leiomyosarcoma after failure of prior anthracyclines and ifosfamide: results of a randomized phase II study of two different schedules. J Clin Oncol.

[10] Dhand UK (2006) Clinical approach to the weak patient in the intensive care unit. Respir Care 51:1024–1040; discussion 1040–1021 16934166

[11] Fetterly GJ, Owen JS, Stuyckens K, Passarell JA, Zannikos P, Soto-Matos A, Izquierdo MA, Perez-Ruixo JJ (2008). Semimechanistic pharmacokinetic/pharmacodynamic model for hepatoprotective effect of dexamethasone on transient transaminitis after trabectedin (ET-743) treatment. Cancer Chemother Pharmacol.

[12] Grosso F, D’Incalci M (2011). Problems in dealing with very rare adverse effects of new anticancer drugs: the example of trabectedin. Tumori.

[13] Grosso F, Dileo P, Sanfilippo R, Stacchiotti S, Bertulli R, Piovesan C, Jimeno J, D’Incalci M, Gescher A, Casali PG (2006). Steroid premedication markedly reduces liver and bone marrow toxicity of trabectedin in advanced sarcoma. Eur J Cancer.

[14] Khan FY (2009). Rhabdomyolysis: a review of the literature. Neth J Med.

[15] Kumar AA, Bhaskar E, Palamaner Subash Shantha G, Swaminathan P, Abraham G (2009). Rhabdomyolysis in community acquired bacterial sepsis—a retrospective cohort study. PLoS ONE.

[16] Monk BJ, Herzog TJ, Kaye SB, Krasner CN, Vermorken JB, Muggia FM, Pujade-Lauraine E, Lisyanskaya AS, Makhson AN, Rolski J, Gorbounova VA, Ghatage P, Bidzinski M, Shen K, Ngan HY, Vergote IB, Nam JH, Park YC, Lebedinsky CA, Poveda AM (2010). Trabectedin plus pegylated liposomal doxorubicin in recurrent ovarian cancer. J Clin Oncol.

[17] Pasternak RC, Smith SC, Bairey-Merz CN, Grundy SM, Cleeman JI, Lenfant C (2002). ACC/AHA/NHLBI clinical advisory on the use and safety of statins. Circulation.

[18] Paz-Ares L, Lopez-Pousa A, Poveda A, Balana C, Ciruelos E, Bellmunt J, Del Muro JG, Provencio M, Casado A, Rivera-Herrero F, Izquierdo MA, Nieto A, Tanovic A, Cortes-Funes H, Buesa JM (2010) Trabectedin in pre-treated patients with advanced or metastatic soft tissue sarcoma: a phase II study evaluating co-treatment with dexamethasone. Invest New Drugs (in press) 10.1007/s10637-010-9561-9 20960029

[19] Perez-Ruixo JJ, Zannikos P, Hirankarn S, Stuyckens K, Ludwig EA, Soto-Matos A, Lopez-Lazaro L, Owen JS (2007). Population pharmacokinetic meta-analysis of trabectedin (ET-743, yondelis((R))) in cancer patients. Clin Pharmacokinet.

[20] Ryan DP, Supko JG, Eder JP, Seiden MV, Demetri G, Lynch TJ, Fischman AJ, Davis J, Jimeno J, Clark JW (2001). Phase I and pharmacokinetic study of ecteinascidin 743 administered as a 72-hour continuous intravenous infusion in patients with solid malignancies. Clin Cancer Res.

[21] Singh D, Chander V, Chopra K (2005). Rhabdomyolysis. Methods Find Exp Clin Pharmacol.

[22] Villalona-Calero MA, Eckhardt SG, Weiss G, Hidalgo M, Beijnen JH, van Kesteren C, Rosing H, Campbell E, Kraynak M, Lopez-Lazaro L, Guzman C, Von Hoff DD, Jimeno J, Rowinsky EK (2002). A phase I and pharmacokinetic study of ecteinascidin-743 on a daily × 5 schedule in patients with solid malignancies. Clin Cancer Res.

[23] Weibrecht K, Dayno M, Darling C, Bird SB (2010). Liver aminotransferases are elevated with rhabdomyolysis in the absence of significant liver injury. J Med Toxicol.

[24] Yovine A, Riofrio M, Blay JY, Brain E, Alexandre J, Kahatt C, Taamma A, Jimeno J, Martin C, Salhi Y, Cvitkovic E, Misset JL (2004). Phase II study of ecteinascidin-743 in advanced pretreated soft tissue sarcoma patients. J Clin Oncol.

